# Increased Dementia Risk in Higher‐Functioning Adults with Autism Spectrum Disorder: Insights from 21,648 Adults with Autism Spectrum Disorder

**DOI:** 10.1002/alz70861_108233

**Published:** 2025-12-23

**Authors:** Aishwarya Gautam, David Kaelber, Pamela Davis, Pauline Terebuh, Rong Xu

**Affiliations:** ^1^ Case Western Reserve University School of Medicine, Cleveland, OH USA; ^2^ The MetroHealth System and Case Western Reserve University School of Medicine, Cleveland, OH USA; ^3^ Case Western Reserve University School of Medicine ‐ ‐ Cleveland, OH, Cleveland, OH USA; ^4^ Case Western Reserve University, Cleveland, OH USA

## Abstract

**Background:**

While intellectual disability (ID), which often co‐occurs with autism, is a known risk factor for dementia, the risk of dementia in higher‐functioning adults with ASD remains understudied.

**Method:**

This retrospective cohort study used electronic health records data from the TriNetX analytics platform, which contains over 134 million aggregated, deidentified U.S. records, to construct four cohorts of adults 30 years or older with: ASD only (n = 21,648), ID only (n = 42,343), both ASD and ID (n = 8,252), and neither diagnosis (n = 3,050,072). After propensity‐score matching cohorts for age and dementia risk factors, the risk of dementia after 3 and 10 years was compared by Kaplan‐Meier curves and hazard ratios.

**Result:**

The mean age of the ASD and general population cohorts after matching was 35.6 ± 9.42 and 35.5 ± 9.39, respectively. A new diagnosis of dementia was substantially more common in the population with ASD (HR=5.8; 95% CI: 3.9‐8.5) at 3 years and (HR=5.0; 95% CI: 3.9‐6.3) at 10 years. The HR for the cohort with both ASD and ID (mean age 36.2 ± 9.61 and 36.2 ± 9.62, respectively) was even greater compared to the general population (HR=12.5; 95% CI: 6.1‐25.6) at 3 years and (HR=8.0; 95% CI: 4.4‐14.6) at 10 years.

**Conclusion:**

Our findings suggest that adults diagnosed with ASD may be at higher risk of developing dementia compared to peers without ASD. Smaller studies of ASD patients have also shown an association between ASD alone and ASD with ID and increased dementia risk compared to the general population, but both of less magnitude. These cumulative findings underscore the importance of heightened awareness and earlier dementia screening in autistic adults.

